# Purified Astaxanthin from *Haematococcus pluvialis* Promotes Tissue Regeneration by Reducing Oxidative Stress and the Secretion of Collagen *In Vitro* and *In Vivo*

**DOI:** 10.1155/2020/4946902

**Published:** 2020-08-03

**Authors:** Hsin-Yu Chou, Dik-Lung Ma, Chung-Hang Leung, Chien-Chih Chiu, Tzyh-Chyuan Hour, Hui-Min David Wang

**Affiliations:** ^1^Program in Tissue Engineering and Regenerative Medicine, National Chung Hsing University, Taichung 402, Taiwan; ^2^Division of Biochemistry and Molecular Biology, Graduate Institute of Medicine, College of Medicine, Kaohsiung Medical University, Kaohsiung 807, Taiwan; ^3^Department of Chemistry, Hong Kong Baptist University, Kowloon Tong, Hong Kong SAR, China; ^4^State Key Laboratory of Quality Research in Chinese Medicine, Institute of Chinese Medical Sciences, University of Macau, Macao SAR, China; ^5^Department of Biotechnology, Kaohsiung Medical University, Kaohsiung City 807, Taiwan; ^6^Department of Medical Research, Kaohsiung Medical University Hospital, Kaohsiung City 807, Taiwan; ^7^Graduate Institute of Biomedical Engineering, National Chung Hsing University, Taichung 402, Taiwan; ^8^Department of Medical Laboratory Science and Biotechnology, China Medical University, Taichung City 404, Taiwan; ^9^Graduate Institute of Medicine, College of Medicine, Kaohsiung Medical University, Kaohsiung City 807, Taiwan; ^10^College of Food and Biological Engineering, Jimei University, Xiamen 361021, China

## Abstract

Intracellular reactive apoptosis and reactive oxygen species (ROS) play a crucial role in ultraviolet- (UV-) induced inflammation and aging reaction in human dermal tissues. This study determines the mechanism by which *Haematococcus pluvialis* extracts (HPE) and purified astaxanthin (HPA) to promote skin regeneration in the injured tissue *in vitro* and *in vivo*. The results show that HPE and HPA decrease the DNA damage and promote the secretion of collagen from the human normal fibroblast cell line (Hs68) in a dose-dependent manner. UV irradiation and HPA reduce oxidative stress damage due to phorbol-12-myristate-13-acetate (PMA). When skin cells are injured by free radicals, cells undergo a programmed cellular death. Cellular apoptotic death is determined using annexin V-fluorescein isothiocyanate (FITC)/propidium iodide (PI) double staining to verify that there is no cell membrane asymmetry and that the nuclear membrane is broken. Inflammatory symptoms and apoptotic injuries to experimental rats in a group that is treated with HPA treated are decreased in a dose-dependent manner after UVB exposure (300 mJ/cm^2^) for 15 min *in vivo*, compared to the vehicle control group. These positive results show that HPA repairs UVB-triggered skin tissue injury and aging by conducting electrons out of cells to maintain a low level of oxidative stress so that collagen is synthesized *in vitro* and *in vivo*.

## 1. Introduction

The skin is composed of three layers—the epidermis, the dermis, and the hypodermis—and is the largest organ in the human body [1]. It maintains internal balance and protects the body from mechanical, biological, physical, and chemical damage [2, 3]. Sunburn, aging, and wrinkles result in a loss of dermal tissues, which inhibits the healing process [4, 5]. The skin is the exterior surface of the human body and is easily exposed to ultraviolet (UV) irradiation from sunshine. Phototoxic harm is a common injury that causes many cuticle diseases and accelerates the aging process.

UVB (290-320 nm) is the most common cause of skin photodamage, including inflammation responses, the production of wrinkles, accumulated aging, and skin cancer [6, 7]. UVB provides the energy to the DNA double helix and causes covalent bonds to break and replication errors. After one day of sunshine exposure, each cell can accumulate DNA damage due to hundreds to thousands lesions, which is degraded into 180–200 base pair fragments by endonuclease [8, 9]. Injury to nuclear DNA is also caused by oxidative free radicals (ROS), which can disperse one or two molecular diameters before acting with other molecular components [10–12]. These injuries cause derma cells to become inflamed, red, and swollen, further triggering a series of inflammation pathways that eventually lead to serious skin problems, including collagen loss and aging.

A previous study by the authors shows that the extract of *Haematococcus pluviali* promotes cell proliferation, collagen production, and antioxidative properties [13]. This study uses purified astaxanthin from *H*. *pluviali*, to regenerate damaged skin. *H*. *pluvialis* is a ubiquitous green algae that belongs to the group of algae, *Rhodococcus*. This alga is widely found in places that are suitable for its growth. This species is famous for its high content of the strong antioxidant, astaxanthin, which is important in aquaculture, food, and cosmetics [1]. Their resting cysts often cause the blood-red color that is seen in the bottom of dried-out rock pools and birdbaths. Large amounts of astaxanthin accumulate within the algal cell when the environment renders survival difficult.

This study uses a bright light and high salinity to promote the production of astaxanthin and extracts and purifies it [14, 15]. It has a molecular weight of 596.86 in geometric *trans*- and *cis*-isomers and a chemical formula of C_40_H_52_O_4_. This study extracts and purifies astaxanthin extract (HPE) and purified astaxanthin (HPA) from red microalgae, *H*. *pluvialis*, the *trans* form of which is more thermodynamically stable than the *cis* form [16].

Within the positive therapeutic potential ingredients, HPA and HPE were reported to have inhibitory effects on the expression of inflammation-related proteins in an animal model. It has been found that HPA obviously suppressed nuclear factor-*κ* light polypeptide gene enhancer (NF-*κ*B) signaling and collagen degradation proteins, matrix metalloproteinases (MMPs). The antioxidant activities and free radical scavenging of some agents have been studied [1, 7, 9, 10, 13], and the results of those studies prompted this work to determine whether HPA reduced photodamage injured and downregulated apoptosis-related RNA and proteins. This study finds that HPA is a potential and positive antioxidant to be a tissue regeneration agent.

## 2. Materials and Methods

### 2.1. Reagents

Dimethyl sulfoxide (DMSO), ethylene diamine tetra-acetic acid (EDTA), and trichloroacetic acid were purchased from Sigma-Aldrich Chemical Corporation (St. Louis, MO, USA). The relevant processes for *H*. *pluvialis* extracts (HPE) and purified astaxanthin (HPA) are reported in a previous study by the authors (Trade Wind Biotech Co. Ltd. (Taiwan)) [13]. Dulbecco's modified eagle medium (DMEM), fetal bovine serum (FBS), antibiotics and other culture mediums were obtained from Gibco BRL (Gaithersburg, MD, USA). Antibodies of COX-2, iNOS, MMP-1, ERK1/2, cleavage-caspase-3, caspase-8, and caspase-9 were purchased from IMGNEX (San Diego, CA, USA). A western blot examination device and buffer solutions were acquired from Cell Signaling Technology Company (Beverly, MA, USA). Other chemical reagents and reaction buffers were obtained at the highest available quality and purity.

### 2.2. Cell Culture

The human normal fibroblast Hs68cell line was obtained from American Type Culture Collection (ATCC, Manassas, VA, USA; Hs68 Number: CRL-1635™). This is one of a series of *Homo sapiens* foreskin fibroblast lines that were developed at the Naval Biosciences Laboratory (NBL) in Oakland, CA, USA. The immortalized human keratinocyte cell line (HaCaT) was kindly provided by Dr. Hamm-Ming Sheu of the Department of Dermatology, National Cheng Kung University Hospital, Tainan, Taiwan. HaCaT and Hs68 were incubated in monolayer conditions with 5% CO_2_ and at 37°C in DMEM that was supplemented with 100 U/ml of penicillin, 0.25 *μ*g/ml of amphotericin B, 100 mg/ml of streptomycin, and10% FBS. All cells were cultured in 6 cm dishes and were observed until the cell density reached 70~80% [17].

### 2.3. UVB Irradiation

UVB radiation was produced using a CL-1000 M UV cross linker (UVP, Inc., Upland, CA, USA), which transmits most of its energy between 290 and 320 nm and peaks at 302 nm. The UVB dose was calculated using a UVX Radiometer (UVP, Inc.). HaCaT and HS68 cells were irradiated by UVB light at a dose of 30 mJ/cm^2^, and rats were treated with 300 mJ/cm^2^. The exposure treatments were based on the results of a preliminary test.

### 2.4. Cell Proliferation Assay (MTT Assay)

The ability of HPE and HPA to repair phototoxic injures was determined using 3-(4,5-dimethylthiazol-2-yl)-2,5-diphenyltetrazolium bromide (MTT) assay procedures [18, 19]. Skin cells were cultured in 96-well plates at a density of 1 × 104 cells/well. After 2 hrs of incubation, the medium was removed and another fresh medium containing 10% MTT solution was added into each well. The cells were maintained at 37°C for 4 h, and the supernatant was removed. DMSO was then injected into each plate well. The optical density was measured at 569 nm using a Bio-Rad 96-well microplate spectrophotometer (BioTek Co. CA, USA). The cell growth is calculated as

(1) $$\begin{array}{c} \text{Cell viability}\%=\frac{\left(A_{\text{sample}}-A_{\text{blank}}\right)}{\left(A_{\text{control}}-A_{\text{blank}}\right)}\times 100\%. \end{array}$$

### 2.5. Collagen Measurement

In order to compare the amount of collagen that is secreted from injured Hs68 cells, HPE and HPA that were incubated in 6-well plates were used [4]. The collagen was stained with Picric-Sirius Red dye (Abcam Co., Cambridge, UK). After coculturing with samples for 24 h, the mediums were removed and the cells were washed twice with phosphate-buffered saline (PBS) buffer (67 mM, pH 6.8). 100 *μ*l of 0.1% dye (0.05 g Sirius Red powder per 50 ml picric acid) was added into each well and reacted at room temperature for another hour. The unstained Sirius Red solution was removed and washed five times with 0.1 N hydrochloric acid. The solution to which stain attached was extracted and thoroughly mixed with 100 *μ*l of 0.1 N NaOH at room temperature for 15 min. To quantify the amount of collagen, the absorbance at 540 nm was monitored using a spectrophotometer (BioTek Co.).

### 2.6. Cellular Comet Assay

1% normal melting point agarose was coated on the slides and air dried. After 24 h, agarose at a concentration of 1% 70 *μ*l was added as a supportive layer. This was gently mixed with 30 *μ*l of cell suspension and 70 *μ*l of 1% low melting point agarose at 37°C. The slides were held in lysis buffer at 4°C for 2 h [20]. The denaturation was performed in electrophoretic alkaline buffer (at pH 13.0; 1 mM EDTA, and 300 mM NaOH) at a cold temperature for 20 min. 20 *μ*l of stain was used per slide of acridine orange (2 *μ*g/ml) for analysis using a fluorescent microscope with a camera (TE2000-U; Nikon Co., Tokyo, Japan). The images were quantified using the OpenComet plugin of the ImageJ software and rearranged for ease of reading. A representative, quantified set of images is attached. The graph was generated by analyzing 3 independent calculations in triplicate using the same software plugin.

### 2.7. Analysis of Intercellular Oxidative Stress Using 2′,7′-Dichlorofluorescin Diacetate (DCFDA)Staining

1 × 104 cells Hs68 and HaCaT cells per well were seeded in 96-well plates. After incubation for 24 hrs, the cells were exposed to UVB radiation and treated using HPA at different concentrations. Phorbol-12-myristate-13-acetate (PMA) was used as the negative control. The cells were suspended and stained with DCFDA solution at 5 *μ*M for 30 min. Individual cells were analyzed using a flow cytometer and excited using a 488 nm laser and detected at 535 nm [21].

### 2.8. Annexin V-FITC/PI-Stained Fluorescence Microscopy

Cells were incubated into a 6-well plate at the density of 1 × 10^6^ cells/well. The incubation medium was lopsided and HaCaT and Hs68 cells were mixed with HPA (5-125 *μ*g/ml) or in the same final volume solution alone (the vehicle control group) with 10% FBS. The staining procedure for the sample with annexin is V-FITC (10 *μ*g/ml)/PI (10 *μ*g/ml) The products were examined using a fluorescent microscope [22]. Double staining was used to identify the cell membrane phosphatidylserine externalization and PI uptake. To quantify any apoptosis, ImageJ software was used to measure the intensity of Annexin V-FITC. The results are from three independent experiments (*n* = 3) to confirm repeatability.

### 2.9. Western Blotting

HaCaT and Hs68 cells were cultured with HPA for 24 h, and cell proteins were extracted using a lysis buffer (Thermo Scientific Pierce RIPA Buffer) [22]. The protein in the supernatant was assayed using a bicinchoninic acid (BCA) protein assay kit (Sigma-Aldrich Corp., USA) after the lysates were centrifuged at 12,000 rpm for 30 min. Proteins were taken in equal quantities, separated using sodium dodecyl sulfate-polyacrylamide gel electrophoresis (SDS–PAGE) on 12% gel, and electrotransferred to a polyvinylidene difluoride (PVDF) membrane. The transfer film was gently removed from the wet transfer tank and semidry transfer slot, and the membrane was blocked using the TBS buffer and 0.1% Tween (TBST) 20 for 1 h. The products were rinsed using 1x TBST to eliminate any traces of skim milk. In each case, the PVDF membrane was incubated with a corresponding primary antibody and washed twice with the TBST buffer. It was then dipped into horseradish peroxidase- (HRP-) conjugated secondary antibodies against the corresponding primary antibody. The samples were then treated with enhanced chemiluminescence (ECL) detection reagents and exposed to X-ray film for specified times to detect bands (PerkinElmer, ECL1 : ECL2 = 1 : 1). Stained blots were visualized using a commercially available imaging system (Thermo Fisher Scientific Co.).

### 2.10. In Vivo Animal Experiment

The use of animals complied with the American Physiology Society's Use of Animals Guiding Principles and was approved by the Kaohsiung Medical University, National Chung Hsing University and Use Committee (KMU104002, KMU105036, and NCHU-IACUC 105-141). Male Wistar rats (255–290 g) were used for animal experiments, and photographs were taken and surgery performed under isoflurane anesthesia [3]. The rats were housed in Plexiglas cages in a temperature-controlled isolated room (22 ± 1°C) on a light/dark (12 h/12 h) schedule and had free access to water and food. Twenty-four rats were randomly divided into 4 groups (*n* = 6, in each group): one vehicle control group, one UV exposure only group, and two HPA-treatment groups. Following anesthetization, dorsal hairs were shaved using an electric razor and were illuminated under UVB 300 mJ/cm^2^ per day (cover eyes, 0.3 W/cm^2^ for 15 min) at the end of 8 weeks. After 24 h, the back skin damage model was completed, and different doses of HPA were injected intraperitoneally.

### 2.11. Statistical Analysis

The difference between the vehicle control and HPA-treated cells was determined using a Student *t*-test for *in vitro* examinations. A one-way ANOVA was used to make a statistical comparison between the vehicle control and experimental groups for *in vivo* assays. A value of *p* < 0.05 denotes statistical significance.

## 3. Results and Discussion

### 3.1. Cell Viability Is Regenerated by Treatment Using HPE and HPA in a Photodamage Skin Model

**Figure 1:**
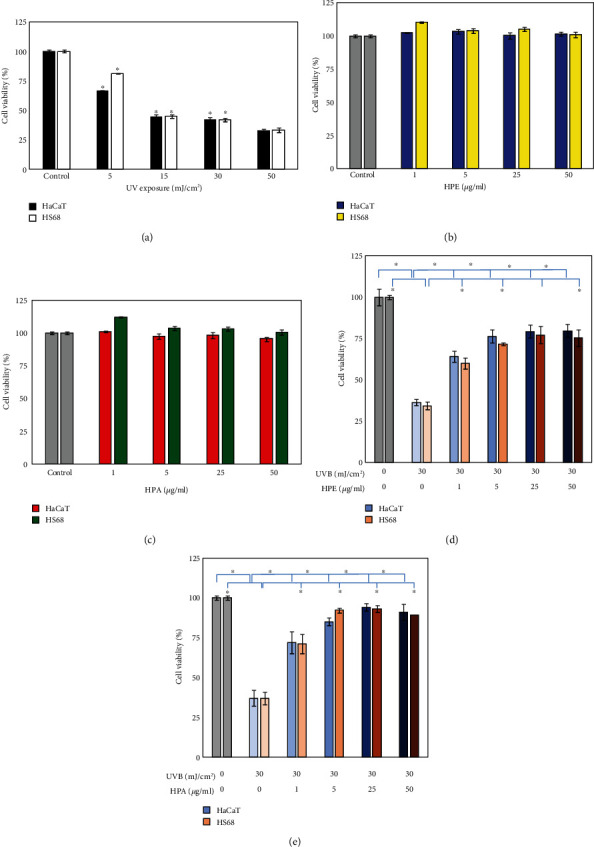
Cell viability of UVB with HPE and HPA 24 hr treatments in a MTT assay. (a) Human skin HaCaT and Hs68 cells were seeded in 96-well microliter plates at a density of 1 × 104 cells/well and exposed to UVB at 5, 15, 30, and 50 mJ/cm^2^. Cells were treated with 1, 5, 25, 50, and 100 *μ*g/ml of (b) HPE and (c) HPA for 24 hrs. Cells were exposed to 5, 15, 30, and 50 mJ/cm^2^ and treated with 1, 5, 25, 50, and 100 *μ*g/ml of (d) HPE and (e) HPA for 24 hrs. The data is shown as a mean value ± standard deviation (SD) for three independent experiments: ^∗^*p* < 0.01.

The skin is the organ that is exposed to the external environment for the longest time. It is often damaged by chemical and physical events such as bacterial infection, environmental pollution (particulate matter, PM_2.5_), and UV light because free radicals are produced, which age skin. Scavenging free radicals increases dermal health and heals inflammation. This study purifies HPA from HPE (Figure S1) and uses it to repair the damage that is caused by free radicals. UVB increases oxidative stress for skin cells and was used to induce cellular damage. To determine the degree of injury to human epidermal and dermal cells due to UVB irradiation, two cells were exposed to periods of UVB radiation (5–30 mJ/cm^2^). Under 5 mJ/cm^2^ UVB stimulation, more than 70% of HaCaT and Hs68 cells survive. Under 30 mJ/cm^2^ UVB, more than 50% of cells die and cell viability is only 40% (Figure 1(a)).

Cellular security, a sensitivity test, and an *in vivo* allergy examination are necessary [23, 24]. To confirm that HPE and HPA is not toxic for normal skin cells, the cytotoxicity of two cells was determined by incubating 0, 1, 5, 25, and 50 *μ*g/ml samples for 24 h periods. The test samples do not induce cytotoxic effects up to 25 *μ*g/ml, and a high dose of 50 *μ*g/ml gives a survival rate of more than an 90% te (Figures 1(b) and 1(c)). After treatment with HPE and HPA, there is a 30% survival rate for 1 *μ*g/ml and a 65% increase at 25 *μ*g/ml. HPE and HPA successfully reverse damage due to UVB radiation in HaCaT and Hs68 cells (Figures 1(d) and 1(e)).

### 3.2. The Inhibition of Intracellular ROS Production by HPA

**Figure 2:**
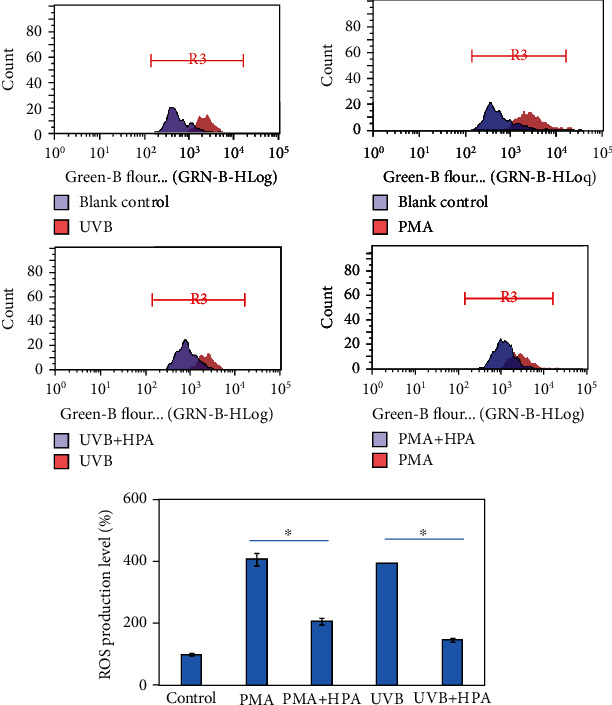
The DCFDA assay results show that HPA scavenges ROS production in skin cells. The data is presented as mean value ± SD for three independent experiments: ^∗^*p* < 0.01.

Figure 1 shows that HPE reduces the damage that is caused by UVB, and HPA is more effective. Many studies show that ROS attacks cells via different mechanisms, including peroxidation of the cell membrane, denaturing of the protein structure, and degradation of DNA [25–27]. Reducing the generation of ROS increases physical well-being. To verify that HPA suppresses ROS, PMA at 20 ng/ml was used as the second negative control and DCFDA was detected in terms of the content of green fluorescence. In Figure 2, flow cytometry shows that both UVB exposure and PMA treatment stimulate an increase in ROS production. Treatment with HPA significantly decreases production of ROS, especially for the UVB+HPA experimental group. The result shows that HPA ROS synthesis is decreased using different resources.

### 3.3. HPA Repaired DNA Damage in Skin Cell

**Figure 3:**
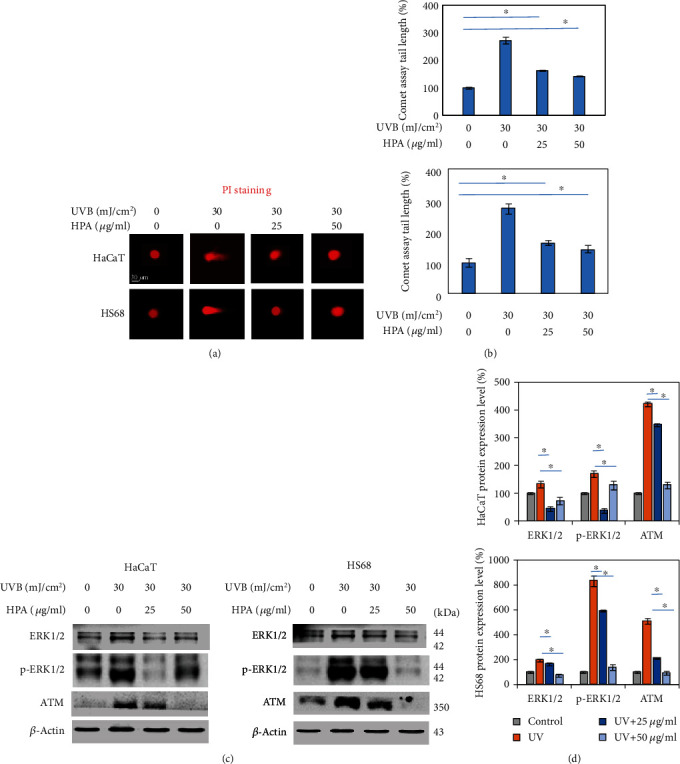
HPA repaired DNA damage in human skin cells. (a) Comet assay was performed to compare DNA damage. PI emits bright red fluorescence in acidic vesicles (fluorescence microscopy, 100x). (b) Quantitative data was presented. (c) The DNA damage-related protein expressions and (d) protein expressions were quantified by using the Image Studio™ Lite software. Results were analyzed as three independent determinations (*n* = 3) to confirm repeatability. *β*-Actin was used as an internal control to ensure equal loading standard. ^∗^*p* < 0.01.

If UVB induces the generation of ROS, ROS degrades the DNA double helix so the strand breaks. A comet assay was used to detect DNA damage. Hs68 and HaCaT cells were irradiated with UVB at 30 mJ/cm^2^ and treated with HPA for 24 hrs. Figures 3(a) and 3(b) show that DNA tails are significantly reduced in a dose-dependent manner using PI staining. The result shows that HPA heals DNA damage after UVB exposure by reducing breakage of the DNA double strand. The mechanisms by which HPA affects DNA repair-related proteins. ATM serine/threonine kinase (ATM) is a protein kinase and is activated by DNA repair detection mechanism to phosphorylate the downstream proteins, so the DNA cell cycle arrests/repairs inflammation and apoptosis reactions [28]. In molecular biology, extracellular signal-regulated kinases (ERKs) express intracellular protein kinase signal molecules, which regulate mitosis, meiosis, and postmitotic activation in differentiated cells [29]. ERK phosphorylation is further triggered by ATM to induce a downstream inflammatory signal pathway. The results of this study show that the protein expression levels for ATM and ERK are decreased in two cell lines in a dose-dependent manner for HPA, as shown in Figures 3(c) and 3(d). The sample reduces DNA injuries and inhibits protein expression.

### 3.4. Collagen Production Diction in Sirius Red Assay

**Figure 4:**
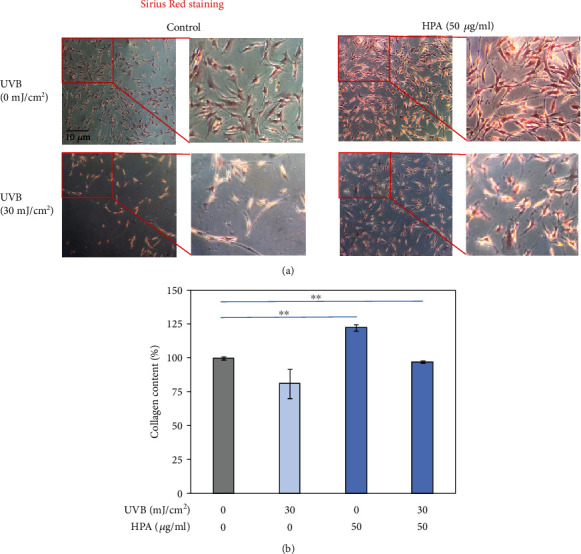
Collagen production of Hs68 cells with HPA treatments in Sirius Red assay. (a) In the HPA-treated group, the collagen content of Hs68 increased in the UVB photo damage model. (b) The quantitative data was presented. The data represented mean values ± SD of three independent experiments performed; *n* = 3, ^∗^*p* < 0.01.

An extracellular matrix (ECM) is a cell-external three-dimensional macromolecule network that is composed of glycoproteins, enzymes, and collagens and enables biochemical and structural maintenance of nearby cells. ECM is a key to cellular adhesion, communication between cells, and cell differentiation [4]. When cells are damaged, they become inflamed and secrete related proteins that decompose ECM and decrease the total collagen amount. Collagen is the most abundant protein in ECM and it is also the most plentiful protein within the human body and forms around ninety percent of the bone matrix protein. Collagens usually occur as fibrillar proteins in ECM and provide a shape skeleton for resident neighbor cells. To determine the change in collagen secretion after skin cells are injured, Sirius Red dye (C_45_H_26_N_10_Na_6_O_21_S_6_, Abcam Co.) was used to mark collagen in red [13].  Figure 4 compares the collagen content after treatment to UVB-injured cells. It is seen that HPA reverts to the collagen production. Collagen production is increased after 24 hr treatment with HPA, so HPA regenerates damaged dermal cells.

### 3.5. The Effect of HPA on the Apoptosis of UVB-Exposed HaCaT and Hs68 Cells

**Figure 5:**
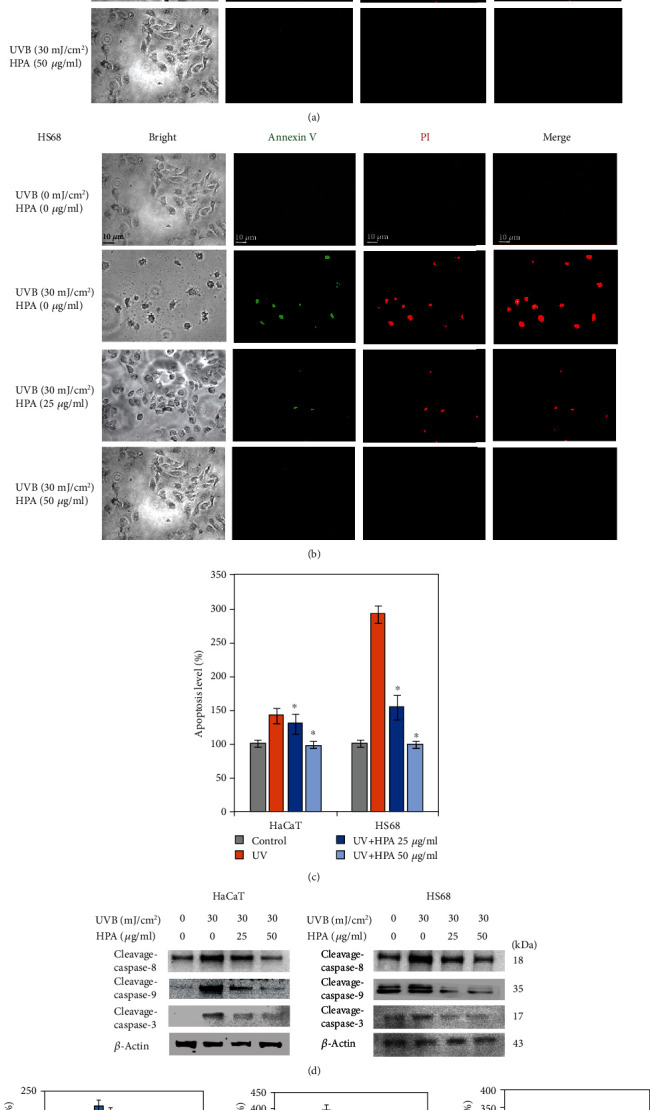
PI/annexin V staining assay with HaCaT and Hs68 cells. (a) HaCaT and (b) Hs68 cells, annexin V fluorescent green in nuclei and cytosol, and PI emits bright red fluorescence in acidic vesicles (fluorescence microscopy, 100x). (c) The stained quantitative image data was presented. (d) Western blot was performed to compare in vitro protein expressions. HPA reduced the apoptosis-related protein levels in both cell lines. (e) Protein expressions were quantified by using the Image Studio™ Lite software. Results were analyzed as three independent determinations (*n* = 3) to confirm repeatability; ^∗^*p* < 0.01.

Fluorescence staining was used to assess the cytotoxic effect on cellular death due to UVB exposure. Annexin V-FITC/PI staining was used to detect apoptosis, and all images were observed under green fluorescence for cellular membranes and red photos for the nucleolus in both cells. HaCaT and Hs68 cells were treated with HPA after exposure to UVB for 90 secs. The manifestation of apoptosis was determined using a cell-based examination under a fluorescent microscope with 400x and the results are shown in Figures 5(a) and 5(b). The ratio of stained cells decreases in a dose-dependent manner from 0 to 50 *μ*g/ml after 24 hrs incubation and apoptotic reactions are reduced. The results show that the UVB-induced lethal apoptotic reactions are significantly inhibited by HPA and both cells recover and survive.

It is reported that UV radiation induces sunburned cellular apoptosis [30]. Apoptosis due to UV is affected by different signaling pathways, which rely on UV intensity and cell type. Caspases are a large family of protease enzymes that significantly affect programmed cellular death, including inflammation, autophagy, pyroptosis, apoptosis, and necroptosis [22, 31]. Normally, caspases are in the inactive stage as prodomain enzymes and then undergo proteolytic courses in conserved aspartic residues to generate two heterosubunits that dimerize the active protease form. Active caspase-8 is essential to the apoptosis execution-phase because of the extrinsic stress that is induced by FasL, FasR, and a variety of apoptotic stimuli. Cleavage-caspase-9 is activated by intrinsic stress and executes a downstream executioner to initiate an apoptotic pathway. Active caspase-3 activates and cleaves caspase-6 and caspase-7 and is processed and operated by caspase-8 and caspase-9. To determine the role of caspases in the apoptosis of HaCaT and Hs68 cells, a western blot assay was conducted and the results are shown in Figures 5(c) and 5(d). These molecular proteins that are associated with apoptosis show notable alterations due to exposure to UVB, including increased cleavage enzymes in caspase-8, caspase-9, and caspase-3. HPA treatment decreases these caspases and suppresses the apoptosis mechanisms.

### 3.6. Relieve Skin Inflammation and ROS Accumulation Induced by UVB Irradiation In Vivo

**Figure 6:**
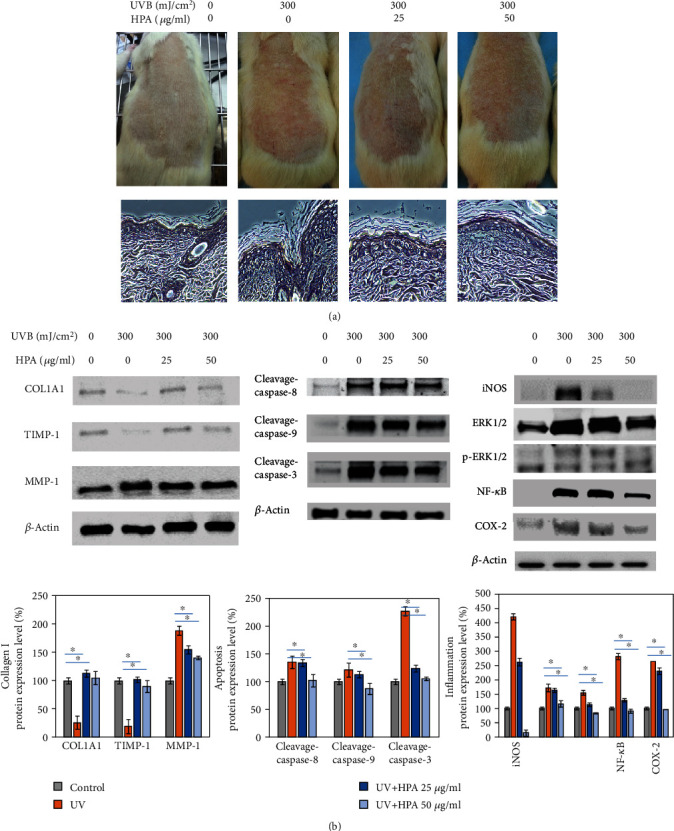
The inflammation characterization after UV irradiation in vivo. (a) The red inflamed and swelling of the rat back skin was observed and evaluated after UV exposure. (b) Western blot was performed to compare protein expressions in vivo. HPA altered the inflammation-related protein levels, apoptosis-related protein expressions, and MMP levels. Cells were harvested from each skin sample, and associated proteins were measured according to Materials and Methods. Protein expressions were quantified by using the Image Studio™ Lite software. Results were analyzed as three independent determinations (*n* = 3) to confirm the repeatability. *β*-Actin was used as an internal control to ensure equal loading standard; ^∗^*p* < 0.01.

This study uses HaCaT and Hs68 cells for UVB experiments, but two cells are not completely representative of the skin because neither cell exhibits a pigment response to the absorption of UVB against oxidative stress *in situ*. Therefore, the *in vitro* assessment on human normal dermal cells is accompanied by an *in vivo* rat model as a more physiologically relevant method to determine the protection mechanisms for HPA [32]. UVB exposure at 300 mJ/cm^2^ for 15 min causes an obvious physical feature of a red edema on the surface of Wistar rats, as seen in Figure 6(a).

In order to assess the effect of HPA on a photodamage repair model, samples harvested from rats' back skin were stained by hematoxylin-eosin staining (H&E) staining after 8 weeks of exposure under UVB administration. We observed that the epidermis of the UVB irradiation group was thicker than the control group, and the dermal cells were in a chaotic state. The sizes of hair follicles were enlarged, and the sweat glands showed abnormal phenomenon in Figure 6(a) of the UVB irradiation group. The above negative consequences were obviously decreased in the HPA-treated group.

We confirmed that UVB stimulation caused inflammation happening and seriously affect collagen production in vitro. HPA alleviated these symptoms through multiple pathways, such as reducing oxidative stress and promoting DNA repair. To observe the effect on animal experiments, we obtained proteins from the rat back skin tissues. The data presented that the content of collagen I had a significant increase after treated with HPA. In addition to the performance of collagen I, we also examined the related molecular signal variety. We found that MMPs were downregulated by HPA treatment, which meant that the inflammation caused by UVB irritation eventually induce the increase of MMPs to lead collagen degradation and finally destroyed the integrity of the dermal tissue. This unsatisfied results were improved after HPA treatment at a dosage of 30 *μ*g/ml. When HPA is injected intraperitoneally, the red swollen area noticeably decreases in size, compared with the UVB only group. There is a marked improvement in the back skin following UVB injury after 1 week, as seen in photographs.

The inflammation symptoms reduce significantly, compared to the vehicle control, so damaged skin tissues are repaired due to treatment with HPA. An irritated itch from a foreign object underneath or upon the rat skin causes the rat to try to remove it. An itch signal creates a scratch and bite reflex, which allows the rat to identify the affected inflammatory site. To reduce the itching sensation, the rat scratches. HPA suppresses the itch mechanism in rats by decreasing the frequency of scratching and biting in fights. Anti-itch mechanisms will be the subject of future study.

A sustained inflammation is not conducive to the repair of injured cells, tissues, or organs. ROS plays a vital role in UVB-triggered inflammatory responses in the skin [33]. Nitric oxide (NO) is a critical molecular signal that acts as a retrograde neurotransmitter and is involved in neural development and angiogenesis. Nitric oxide synthases (NOS) are formed in the production of NO catalyzed from L-arginine. NO is mediated in humans by the calcium-calmodulin-controlled isoenzyme endothelial NOS and neuronal NOS. The inducible nitric oxide synthase isoform (iNOS) is a free radical with an unpaired electron that acts as an immune defense mechanism [34].

The effect of HPA on inflammatory reactions in the skin tissue from the rat epidermal skin was tested.  Figure 6(b) shows the results for rats that are treated with HPA after irradiation with UVB. The skin tissue result shows significant recovery from the decrease in ROS, and the UVB-induced iNOS protein content is also reduced. After treatment with HPA at 25 and 50 *μ*g/ml, COX-2 expression levels decrease, compared to the vehicle control group. The relief of inflammation and the increased thickness of the epidermis showed that HPA can be used to treat photodamage.

Rats were treated with HPA at concentrations of 25 and 50 *μ*g/ml for 24 hrs after UVB exposure at 300 mJ/cm^2^ for 15 min to determine the apoptotic response. Compared to the group that is exposed to UVB, there is a smaller increase in apoptosis-related protein in the HPA-treated group. Cleavage-caspase-8, caspase-9, and caspase-3 proteins play an important role in apoptosis [35]. UVB irradiation results in greater expression of these three proteins, and treatment with HPA significantly inhibits these UV radiation-induced changes in the expression of apoptosis-related proteins, as shown in Figures 6(c) and 6(d).

## 4. Conclusion

**Figure 7:**
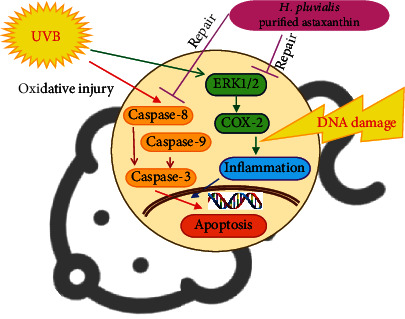
A schematic representation of the signaling pathway that involved the repair of oxidative damage to UVB-induced skin cells by HPA.

The results show that HPA that is purified from *H*. *pluvialis* can regenerate injured skin that is subjected to UVB-induced photodamage *in vitro* and *in vivo* (Figure 7). This study shows the recuperative effect of HPA on HaCaT and Hs68 cells through the reduction in DNA damage and the downregulation of caspase and ERK proteins. HPA decreases ROS accumulations, which subsequently induce inflammation, so it inhibits UVB-induced apoptosis *in vivo* and reduces skin aging.

## Data Availability

The authors confirm that all data is fully available without restriction. All relevant data is described within the paper.

## Supplementary Materials

Supplementary Materials Figure S1: HPE was extracted to obtain HPA. According to high-performance liquid chromatography (HPLC) procedure, HPA contained high-purity astaxanthin over 95%.
