# Supplementary material for: Purified Astaxanthin from Haematococcus pluvialis Promotes Tissue Regeneration by Reducing Oxidative Stress and the Secretion of Collagen In Vitro and In Vivo
Source: Oxid Med Cell Longev. 2020 Aug 3;2020:4946902. doi: 10.1155/2020/4946902 (PMC7424503; doi:10.1155/2020/4946902)
Supplement: Supplementary Materials — Figure S1: HPE was extracted to obtain HPA. According to high-performance liquid chromatography (HPLC) procedure, HPA contained high-purity astaxanthin over 95%. [file 4946902.f1.doc]

**Supporting Information**

**Purified astaxanthin from *Haematococcus pluvialis* promotes tissue regeneration via oxidative stress reduction and collagen secretion stimulation *in vitro* and *in vivo***

Hsin-Yu Chou,^1,2^ Chung-Hang Leung,^3^ Dik-Lung Ma,^4^ Tzyh-Chyuan Hour,^2,*^ and Hui-Min David Wang ^1,5,6,7,8,*^

^1^ *Ph.D. Program in Tissue Engineering and Regenerative Medicine, National Chung Hsing University, Taichung 402, Taiwan*

*^2^ Division of Bio chemistry and Molecular Biology, Graduate Institute of Medicine, College of Medicine, Kaohsiung Medical University, Kaohsiung 807, Taiwan*

*^3^ State Key Laboratoryof Quality Research in Chinese Medicine, Institute of Chinese Medical Sciences, University of Macau, Macao, China*

*^4^ State Key Laboratoryof Quality Research in Chinese Medicine, Institute of Chinese Medical Sciences, University of Macau, Macao, China*

^5^ *Graduate Institute of Biomedical Engineering, National Chung Hsing University, Taichung 402, Taiwan*

*^6^* Graduate Institute of Medicine, College of Medicine, Kaohsiung Medical University, Kaohsiung City 807, Taiwan

*^7^* *Department of Medical Laboratory Science and Biotechnology, China Medical University, Taichung City 404, Taiwan*

*^8^* College of Food and Biological Engineering, Jimei University, Xiamen 361021, PR China

**CORRESPONDENCES**

Hui-Min David Wang, Ph.D.
Professor, Graduate Institute of Biomedical Engineering, National Chung Hsing University, Taichung, Taiwan
No.145, Xingda Rd., South Dist., Taichung City 402, Taiwan
Mobil: 886-935753718
TEL: 886-4-22840733#651
Fax: 886-22852242
E-mail: [davidw@dragon.nchu.edu.tw](mailto:davidw@dragon.nchu.edu.tw)

Tzyh-Chyuan Hour
Professor, Division of Biochemistry and Molecular Biology, Graduate Institute of Medicine, College of Medicine, Kaohsiung Medical University, Kaohsiung 807, Taiwan
Mobil: 886-922660639
TEL: 886-7-3121101#2138
Fax: 886-7-3223075
E-mail: [cliff@cc.kmu.edu.tw](mailto:cliff@cc.kmu.edu.tw)


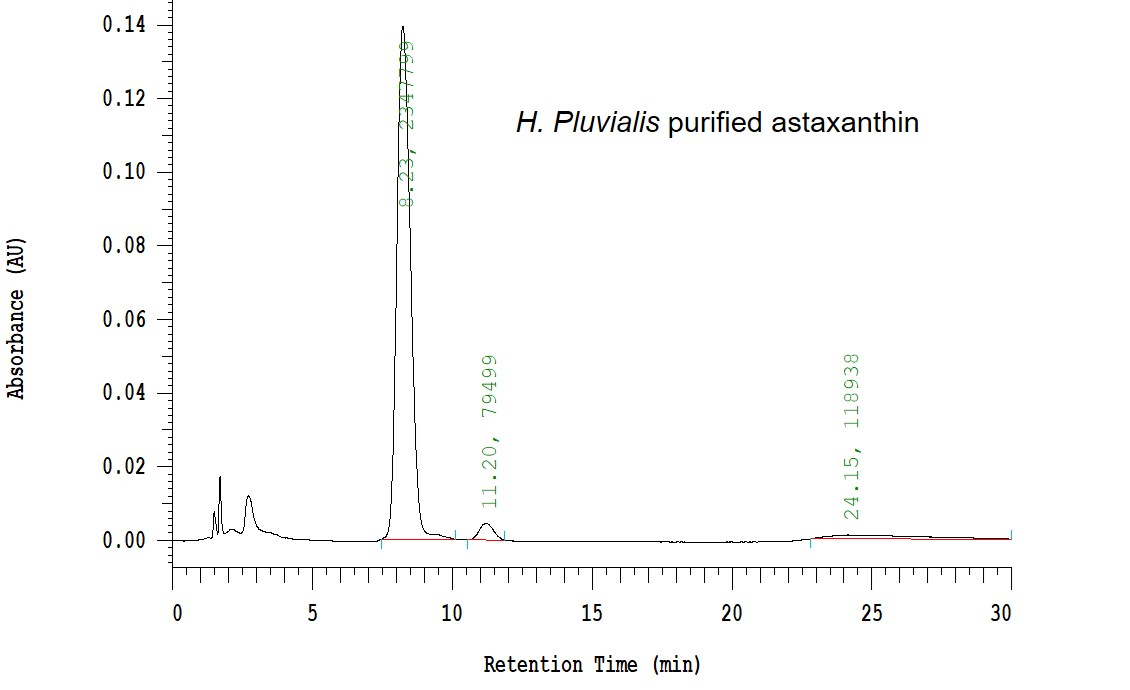


**Figure S1.** HPE was extracted to obtain HPA. According to high performance liquid chromatography (HPLC) procedure, HPA contained high-purity astaxanthin over 95%.
